# Expression and function of junctional adhesion molecule-C in human and experimental arthritis

**DOI:** 10.1186/ar2223

**Published:** 2007-07-05

**Authors:** Gaby Palmer, Nathalie Busso, Michel Aurrand-Lions, Dominique Talabot-Ayer, Véronique Chobaz-Péclat, Claudia Zimmerli, Philippe Hammel, Beat A Imhof, Cem Gabay

**Affiliations:** 1 Division of Rheumatology, Department of Internal Medicine, University Hospital, 26 avenue Beau-Séjour, 1211 Geneva 14, Switzerland and Department of Pathology and Immunology, University of Geneva School of Medicine, 1 rue Michel-Servet, 1211 Geneva 4, Switzerland; 2 Division of Rheumatology, Department of Medicine, University Hospital, Nestlé 05-5029, 1011 Lausanne, Switzerland; 3 Department of Pathology and Immunology, University of Geneva School of Medicine, 1 rue Michel-Servet, 1211 Geneva 4, Switzerland

## Abstract

Junctional adhesion molecule-C (JAM-C) is an adhesion molecule involved in transendothelial migration of leukocytes. In this study, we examined JAM-C expression in the synovium and investigated the role of this molecule in two experimental mouse models of arthritis. JAM-C expression was investigated by reverse transcriptase-polymerase chain reaction and immunohistochemistry. The effects of a monoclonal anti-JAM-C antibody were assessed in antigen-induced arthritis (AIA) and K/BxN serum transfer-induced arthritis. JAM-C was expressed by synovial fibroblasts in the lining layer and associated with vessels in the sublining layer in human and mouse arthritic synovial tissue. In human tissue, JAM-C expression was increased in rheumatoid arthritis (RA) as compared to osteoarthritis synovial samples (12.7 ± 1.3 arbitrary units in RA versus 3.3 ± 1.1 in OA; *p *< 0.05). Treatment of mice with a monoclonal anti-JAM-C antibody decreased the severity of AIA. Neutrophil infiltration into inflamed joints was selectively reduced as compared to T-lymphocyte and macrophage infiltration (0.8 ± 0.3 arbitrary units in anti-JAM-C-treated versus 2.3 ± 0.6 in isotype-matched control antibody-treated mice; *p *< 0.05). Circulating levels of the acute-phase protein serum amyloid A as well as antigen-specific and concanavalin A-induced spleen T-cell responses were significantly decreased in anti-JAM-C antibody-treated mice. In the serum transfer-induced arthritis model, treatment with the anti-JAM-C antibody delayed the onset of arthritis. JAM-C is highly expressed by synovial fibroblasts in RA. Treatment of mice with an anti-JAM-C antibody significantly reduced the severity of AIA and delayed the onset of serum transfer-induced arthritis, suggesting a role for JAM-C in the pathogenesis of arthritis.

## Introduction

The recruitment of leukocytes to inflamed tissues is a highly regulated multistep process, which includes leukocyte rolling on the vascular endothelium, activation of leukocytes and subsequent firm adhesion to endothelial ligands, transendothelial migration from the vascular lumen into the surrounding tissue, and migration of inflammatory cells through the tissue in response to chemokine gradients [1,2]. The successive events in this cascade are mediated by coordinated interaction of adhesion molecules expressed by leukocytes, endothelial cells, and the surrounding tissues. In particular, endothelial transmigration involves the interaction of leukocytes with adhesion molecules expressed on the endothelial cell surface, whereas their retention likely involves interaction with adhesion molecules present on different cell types residing within the target tissue.

Transendothelial migration of leukocytes involves several endothelial adhesion molecules regulating the paracellular trafficking, such as CD99, platelet endothelial cell adhesion molecule-1 (PECAM-1), or the junctional adhesion molecules (JAMs) [3-6]. The JAM protein family consists of three members called JAM-A, JAM-B, and JAM-C, which are immunoglobulin (Ig) superfamily molecules with two extracellular Ig domains and a short cytoplasmic tail, ending with a PDZ-binding motif, involved in cytoskeletal and signal transduction interactions [7]. JAM-C was initially described as an adhesion molecule localized at interendothelial contacts and as an integrin ligand mediating interactions between vascular cells and leukocytes [5,8]. JAM-C is also expressed in mesenchymal and epithelial cells, suggesting that in addition to its role in inflammatory cell recruitment, it might contribute to the retention of leukocytes within inflamed tissues [9,10].

Soluble JAM-C has been demonstrated to inhibit neutrophil transmigration both *in vitro *and *in vivo *[6]. Similarly, monoclonal antibodies directed against JAM-C reduced the accumulation of leukocytes in alveoli during acute pulmonary inflammation in mice [11], prevented leukocyte influx in a murine model of allergic contact dermatitis [12], and decreased inflammatory cell recruitment and tissue injury in cerulein-induced acute pancreatitis [13].

Uncontrolled activation of leukocytes and endothelial cells is a feature of pathologic chronic inflammation, such as observed in rheumatoid arthritis (RA). The mechanisms regulating recruitment and retention of leukocytes in the joint in experimental models of inflammatory arthritis and the role of various adhesion molecules in human RA are still poorly understood. The aim of the present study was to investigate the role of JAM-C in arthritis. We describe the expression of JAM-C in human and mouse synovium and synovial fibroblasts. Furthermore, we observed that a monoclonal anti-JAM-C antibody decreased the severity of mouse antigen-induced arthritis (AIA) and delayed the onset of K/BxN serum transfer-induced arthritis.

## Materials and methods

### Mice

Male C57BL/6 mice were obtained from Janvier (Le Genest-St-Isle, France) and used between 9 and 11 weeks of age. KRN T-cell receptor transgenic mice, developed in the laboratory of Diane Mathis and Christophe Benoist, were kindly provided by the Institut de Génétique et de Biologie Moléculaire et Cellulaire (Strasbourg, France) [14] and were maintained on a C57BL/6 background (K/B). Progeny bearing the V^β^6 transgenic T-cell receptor were identified by cytofluorometry of peripheral blood lymphocytes using antibodies labeled with anti-CD4 phycoerythrin (clone L3T4; BD Pharmingen, San Diego, CA, USA) and anti-V^β^6 fluorescein isothiocyanate (clone RR4-7; BD Pharmingen). NOD/Lt mice were purchased from The Jackson Laboratory (Bar Harbor, ME, USA). All mice were housed under conventional conditions, and water and standard laboratory chow were provided *ad libitum*. All animal experiments were approved by the Animal Ethics Committee of the Geneva University School of Medicine and by the Geneva Veterinarian Office.

### Antigen-induced arthritis

Mice were injected intradermally at the base of the tail with 100 μg of methylated bovine serum albumin (mBSA) (Fluka, part of Sigma-Aldrich, St. Louis, MO, USA), emulsified in complete Freund's adjuvant (Difco Laboratories Inc., now part of Becton Dickinson and Company, Franklin Lakes, NJ, USA) containing 5 mg/ml *Mycobacterium tuberculosis*. Heat-killed *Bordetella pertussis *organisms (0.2 × 10^9^) (Berna, Bern, Switzerland) were injected intraperitoneally as an additional adjuvant. On day 7, a booster injection of 100 μg of mBSA in incomplete Freund's adjuvant (Becton Dickinson and Company) was given at the base of the tail. On day 21, arthritis was induced by intra-articular injection of 100 μg of mBSA in 10 μl of phosphate-buffered saline (PBS) into the left knee joint of mBSA-immunized mice, the right knee being injected with sterile PBS alone. The monoclonal anti-JAM-C antibody H36 [15] or an isotype-matched control antibody (9B5, rat IgG2a anti-human CD44) was injected (150 μg/mouse, intraperitoneal [i.p.]) 1 hour before intra-articular injection of mBSA into the left knee and of PBS into the right knee. Mice were sacrificed 4 or 8 days after induction of arthritis, the latter group receiving a second injection of antibodies on day 4. The development of arthritis was followed by measuring technetium-99m (Tc) uptake in the knees on days 1, 3, and 7 after intra-articular mBSA injection as previously described [16].

### K/BxN serum transfer-induced arthritis

Arthritic K/BxN mice were obtained by crossing K/B mice with NOD/Lt (N) animals. Arthritic adult K/BxN mice were bled and the sera were pooled. Recipient C57BL/6 mice were injected with pooled serum (100 μl of serum i.p. on days 0 and 6). The monoclonal anti-JAM-C antibody H36 or an isotype-matched control antibody (9B5) was injected (150 μg/mouse, i.p.) 1 hour before the first injection of serum on day 0 and then again on days 4 and 8. Mice were sacrificed on day 13. The development of arthritis was assessed daily, and the severity of arthritis was scored in a blinded fashion for each paw on a 3-point scale, in which 0 = normal appearance, 1 = localized edema/erythema on one digit or over one surface of the paw, 2 = edema/erythema involving more than one surface of the paw, and 3 = marked edema/erythema involving the whole paw. The scores of all four paws were added for a composite score.

### Histological grading of arthritis

At sacrifice, the knees (AIA) or the paws (K/BxN serum transfer-induced arthritis) were dissected and fixed in 10% buffered formalin for 7 days. Fixed tissues were decalcified for 3 weeks in 15% EDTA (ethylenediaminetetraacetic acid), dehydrated, and embedded in paraffin. Sagittal sections (5 μm) of the whole joint were stained with safranin O and counterstained with fast green/iron hematoxilin. Histological sections were graded independently by two observers unaware of the treatment group by using the following parameters. For AIA, synovial membrane thickness, which reflects the degree of synovial inflammation and hyperplasia, was scored on a scale of 0 to 6 (0 = normal thickness to 6 = maximal thickness). Cartilage proteoglycan depletion, reflected by loss of safranin O staining intensity, was scored on a scale of 0 (fully stained cartilage) to 6 (totally unstained cartilage). For the K/BxN serum transfer-induced arthritis, synovial membrane thickness around the ankle, exudates in the ankle region, and ankle edema were scored on a scale of 0 to 3 (0 = normal to 3 = maximal).

### Human synovial tissue samples

Specimens of synovial tissue from osteoarthritis (OA) and RA patients undergoing joint surgery of the knee or the hip were obtained from the Department of Orthopedics of the Centre Hospitalier Universitaire Vaudois (Lausanne, Switzerland). Patients with RA fulfilled at least four of the seven American College of Rheumatology revised criteria for RA. All tissues were cut into small pieces and immediately frozen in precooled hexane and stored at -70°C until use. All subsequent analyses were performed on consecutive cryostat sections (one representative piece analyzed per patient). Samples were obtained after appropriate informed consent, and their use for research was approved by the Ethics Committee.

### Immunohistochemistry

JAM-C expression was studied by immunohistochemistry (IHC) using rabbit polyclonal antibodies against human or murine JAM-C on cryostat sections of human or murine synovial tissues, respectively. Antibodies against mouse and human JAM-C have been previously described [17,18]. Lymphocyte, macrophage, and neutrophil infiltrations into mouse synovium were detected by IHC using anti-CD3, anti-MAC-2, or anti-MPO antibodies, respectively, on paraffin-embedded sections. Briefly, frozen or deparaffinized and rehydrated sections were incubated for 30 minutes at room temperature with 5% BSA and 20% normal serum. Endogenous peroxidase activity was blocked with 3% H_2_O_2 _for 10 minutes. Slides were then overlaid with the primary antibody for 1 hour at room temperature. Bound antibody was visualized using the avidin-biotin-peroxidase complex (Vectastain Elite ABC kit; Vector Laboratories, Burlingame, CA, USA). The color was developed by 3,3'-diaminobenzidine (Sigma-Aldrich) containing 0.01% H_2_O_2_. After extensive washing in water, slides were counterstained with Papanicolaou (Merck AG, Dietikon, Switzerland) and mounted in Merckoglass (Merck AG, Dietikon, Switzerland). Staining specificity was confirmed using preimmune serum (for anti-JAM-C IHC), isotype-matched antibodies (for anti-CD3 and anti-MAC-2 IHC), or matched serum (for anti-MPO IHC) as primary antibodies. An incubation without the first antibody served as a negative control. Infiltration of lymphocytes, macrophages, and neutrophils was assessed by semi-quantitative visual scoring of CD3, MAC-2, and MPO immunostaining in the synovial membrane. For each marker, staining was graded independently by two observers (unaware of animal treatment) on a scale of 0 (no staining at all) to 3 (maximal staining). To estimate JAM-C levels in human synovial tissues, sections were magnified 400 times through a microscope (Olympus, Mont-sur-Lausanne, Switzerland), scanned using a JVC TK-C1381 color video camera (Olympus) and analyzed using Semper 6P image analysis software (Synoptics Ltd, Cambridge, UK). The results were expressed as the ratio between the number of pixels associated to immunoreactive regions and between the number of pixels of the total area examined.

### Culture of human and mouse synovial fibroblasts

Human synovial fibroblasts were isolated by collagenase digestion as reported previously [19]. Murine synovial fibroblasts were prepared from synovial tissue dissected from AIA knee joints. The tissue was finely minced and then digested in 0.1% collagenase (Gibco-BRL, now part of Invitrogen Corporation, Carlsbad, CA, USA) in Dulbecco's modified Eagle's medium (DMEM) for 2 hours at 37°C. Undigested material was removed, and cells were collected by centrifugation. Both human and murine synovial fibroblasts were kept in primary culture, at 37°C, in a humidified atmosphere containing 5% CO_2_, in DMEM supplemented with 10% fetal calf serum and 100 U/ml penicillin and 100 μg/ml streptomycin. Non-adherent cells were removed by repeated washings during cell culture, and cells were used at the third passage.

### Reverse transcriptase-polymerase chain reaction

Total RNA was isolated from human synovial tissue samples, knees of mice with AIA or collagen-induced arthritis [20], paws of mice with K/BxN serum transfer-induced arthritis, and human and mouse synovial fibroblasts using the TRIzol reagent (Invitrogen Corporation). Total RNA (1 to 3 μg) was digested with DNAse I (Promega AG, Wallisellen, Switzerland) and reverse-transcribed using avian myeloblastosis virus reverse transcriptase (RT) (Promega AG) and random hexamer primers. Polymerase chain reaction (PCR) amplification (40 cycles for *JAM-C *and 30 cycles for *β-actin*) was performed using Taq DNA polymerase (Qiagen AG, Hombrechtikon, Switzerland) and the following primers: murine *Jam-C *forward primer 5'-TGC TGC TGC TCT TCA GGG GC-3' and murine *Jam-C *reverse primer 5'-GAC AGG GGT CAC TGG CTT C-3' (GenBank accession number: NM023844), human *JAM-C *forward primer 5'-CTG GGG AAG ACA TCC CTG AAG-3' and human *JAM-C *reverse primer 5'-AGT GCG GAT GTA GTT AAC TCC-3' (GenBank accession number: NM032801), and *β-actin *forward primer 5'-CCAAGGCCAACCGCGAGAAGATGAC-3' and *β-actin *reverse primer 5'-AGGGTACATGGTGGTGCCGCCAGAC-3' (GenBank accession number: M10277). Annealing temperatures were 55°C for *JAM-C *and 60°C for *β-actin*. The absence of DNA contamination in RNA preparations was tested by including RNA samples, which had not been reverse-transcribed, and distilled water was used as a negative control for PCR amplification. The identity of the amplified products was confirmed by DNA sequencing.

### Measurement of serum amyloid A levels

Blood was taken at the end of experiment by cardiac puncture. Serum levels of serum amyloid A (SAA) were determined using a direct enzyme-linked immunosorbent assay (ELISA) as previously described [21]. The detection limit for this test is 13 μg/ml.

### T-cell proliferation assay

Spleen cells were harvested on day 4 after intra-articular mBSA injection and seeded at 4 × 10^5 ^cells per well in 96-well plates in 200 μl of RPMI 1640 medium containing 100 IU/ml penicillin, 100 μg/ml streptomycin, 5 × 10^-5 ^M β-mercaptoethanol, and 1% mouse serum. Cells were incubated at 37°C with 5% CO_2 _for 72 hours without or with 10 μg/ml mBSA or 5 μg/ml concanavalin A (ConA) (Amersham Pharmacia Biotech, now part of GE Healthcare, Little Chalfont, Buckinghamshire, UK). During the final 18 hours of incubation, ^3^H-thymidine was added at 1 μCi/well. Cells were harvested and radioactivity was counted to determine ^3^H-thymidine incorporation into DNA as a measure of cell proliferation.

### Determination of cytokine and antibody production

Spleen cells were harvested on day 4 after intra-articular mBSA injection and cultured for 72 hours without or with 10 μg/ml mBSA or 5 μg/ml ConA. Culture supernatants were harvested, and interferon-gamma (IFN-γ) and interleukin-10 (IL-10) levels were quantified by ELISA by means of a commercial DuoSet ELISA Development System (R&D Systems, Abingdon, UK) for IFN-γ and a BD OptEIA Set (BD Biosciences, Heidelberg, Germany) for IL-10. The detection limits for both tests are 31 pg/ml. Serum levels of total anti-mBSA antibodies were measured as described previously [16].

### Statistical analysis

Significance of differences was calculated by analysis of variance, chi-square test, or Wilcoxon rank sum test as indicated. A difference between experimental groups was considered significant when the *p *value was less than 0.05.

## Results

### Expression of junctional adhesion molecule-C in human and mouse synovial tissues

**Figure 1:**
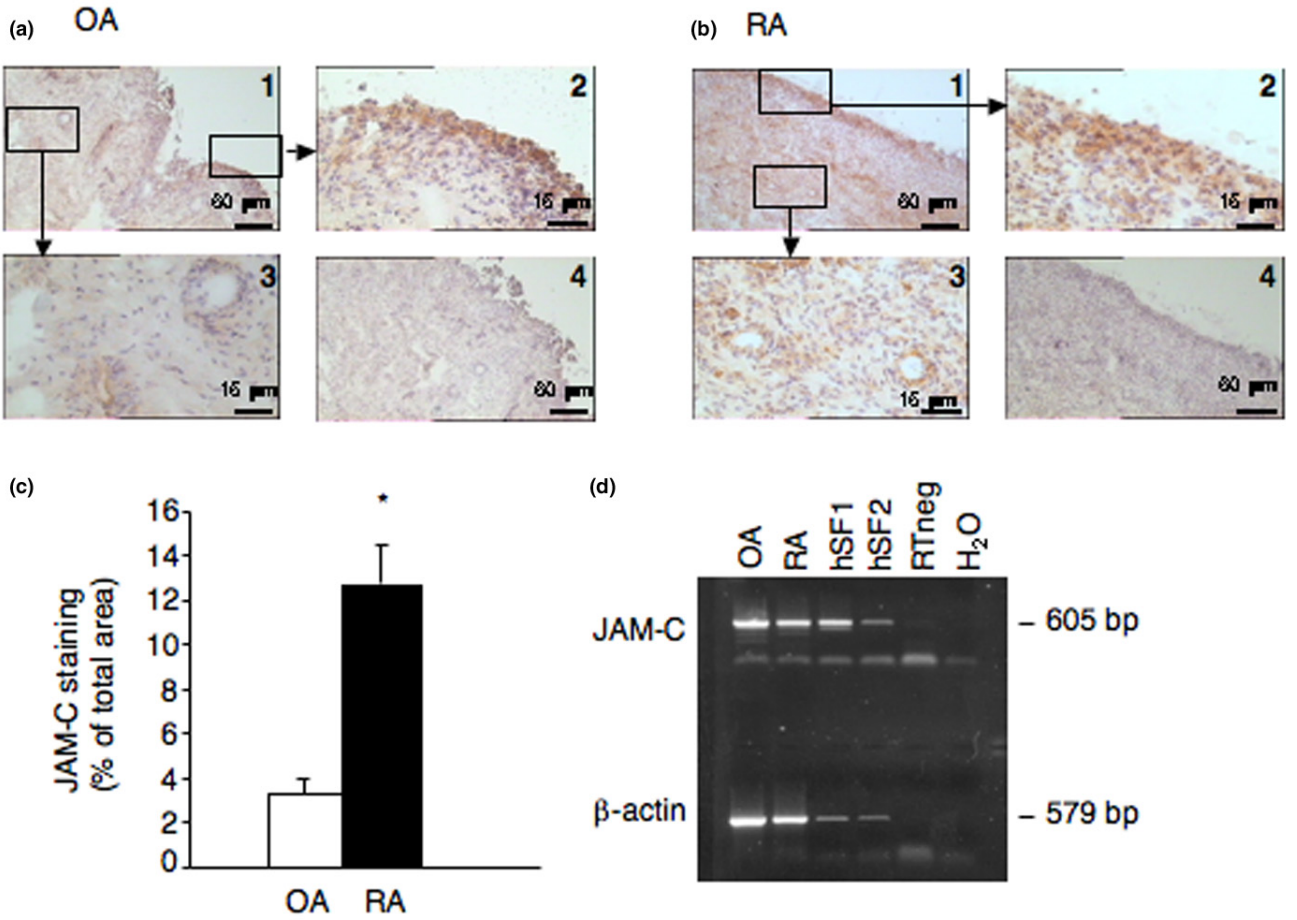
Expression of junctional adhesion molecule-C (JAM-C) in human arthritic synovium. Expression of JAM-C was examined by immunohistochemistry in human osteoarthritis (OA) **(a) **and rheumatoid arthritis (RA) **(b) **synovial tissue. The panels labeled 1 show the original magnification (× 100). The panels labeled 2 show expression of JAM-C in the synovial lining layer (magnification × 400), and panels labeled 3 show JAM-C associated with blood vessels in the sublining (magnification × 400). The panels labeled 4 show negative control sections incubated with preimmune serum. Scale bars = 60 μm (panels 1 and 4) and 15 μm (panels 2 and 3). **(c) **Quantification of JAM-C expression in OA and RA synovial tissues. JAM-C immunohistochemical synovial tissue sections from four different OA and RA patients were scanned, and the surface of immunoreactive areas was determined and expressed as the percentage of the surface of the image examined. Results are expressed as the mean ± standard error of the mean. *p < 0.001 OA versus RA as assessed using the Wilcoxon rank sum test. **(d) **Reverse transcriptase-polymerase chain reaction (PCR) analysis of *JAM-C *mRNA expression in human synovial tissue samples and in cultured human synovial fibroblasts. A representative agarose gel electrophoresis of PCR products is shown. bp, base pairs; H_2_O, polymerase chain reaction negative control; hSF1 and hSF2, human rheumatoid arthritis synovial fibroblast cultures from two different patients used after the third passage; OA, osteoarthritis synovial tissue; RA, rheumatoid arthritis synovial tissue; RT neg, non-reverse-transcribed sample.

We investigated JAM-C expression in human OA and RA synovial samples by IHC using polyclonal antibodies against human JAM-C. Interestingly, expression of JAM-C was mainly found in the lining layer and was associated with vessels in the sublining layer (Figure 1a,b) in OA and in RA synovial biopsies. JAM-C expression, as quantified by histomorphometry, was significantly higher in RA than in OA samples (Figure 1c). RT-PCR analysis showed *JAM-C *mRNA expression in OA and RA synovial biopsies as well as in purified human synovial fibroblasts (Figure 1d), suggesting that these cells account for at least part of the JAM-C expression observed in the lining layer by IHC.

**Figure 2:**
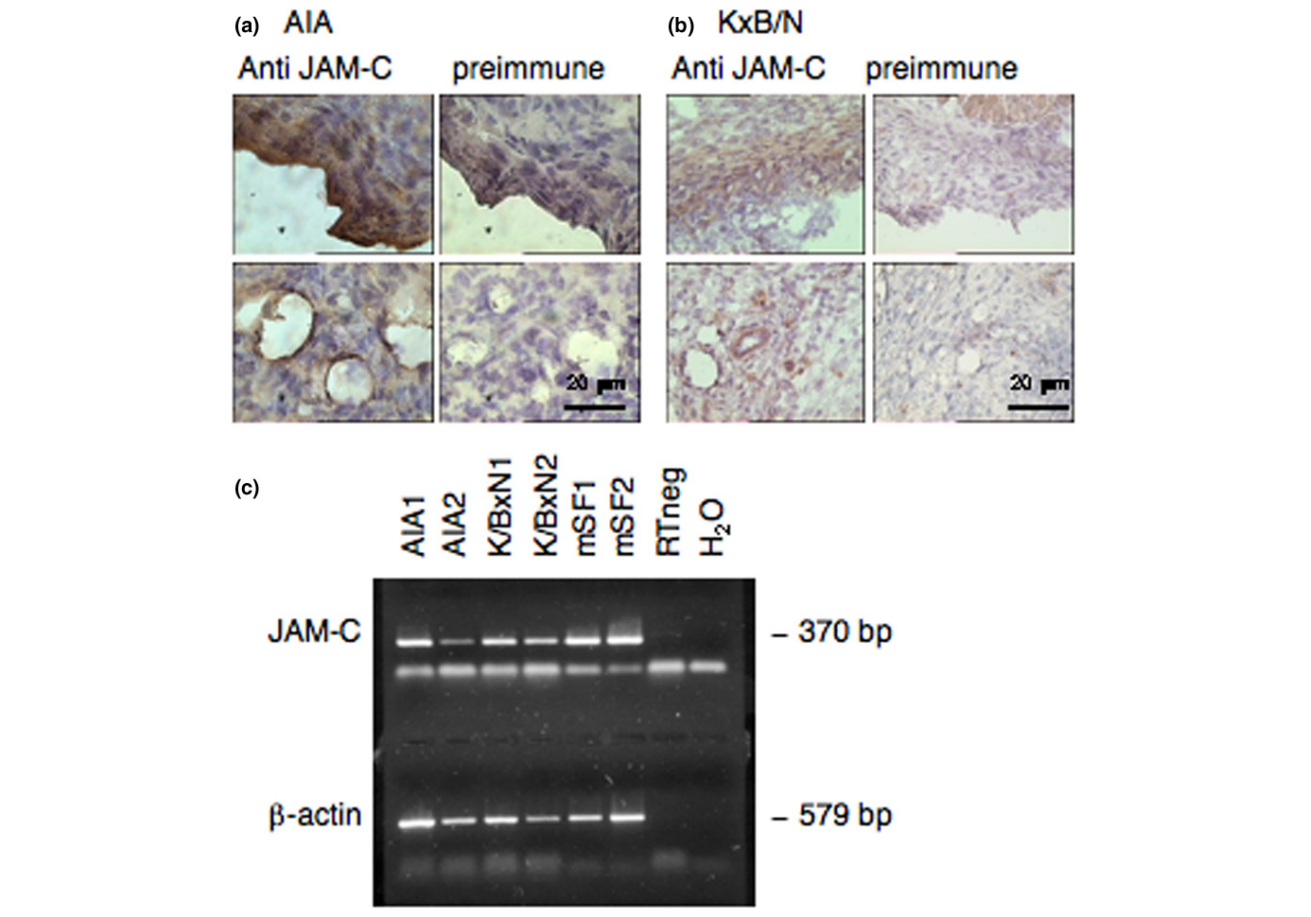
Expression of junctional adhesion molecule-C (JAM-C) in mouse arthritic synovium and synovial fibroblasts. Expression of JAM-C was examined by immunohistochemistry in knee joints of mice with antigen-induced arthritis (AIA) **(a) **and in paws of mice with K/BxN serum transfer-induced arthritis **(b)**. The left panels show expression of JAM-C in the synovial lining layer (upper panel) and associated with blood vessels in the sublining (lower panel). The right panels show negative control sections incubated with preimmune serum. Original magnification × 400 (scale bar = 20 μm). **(c) **Reverse transcriptase-polymerase chain reaction (PCR) analysis of *JAM-C *mRNA expression in mouse synovial tissue samples and in cultured mouse synovial fibroblasts. A representative agarose gel electrophoresis of PCR products is shown. AIA1 and AIA2, antigen-induced arthritis synovial tissue; bp, base pairs; H_2_O, polymerase chain reaction negative control; K/BxN1 and K/BxN2, K/BxN serum transfer-induced arthritis synovial tissue; mSF1 and mSF2, two different mouse synovial fibroblast cultures isolated from antigen-induced arthritis synovial tissue; RTneg, non reverse-transcribed sample.

To confirm our findings and extend our observations to another species, we then studied the pattern of JAM-C expression in two different mouse models of experimental arthritis: AIA and K/BxN serum transfer-induced arthritis (Figure 2a,b). In both cases, IHC using polyclonal antibodies against mouse JAM-C showed expression of JAM-C in a pattern similar to that found in human arthritic tissues. Expression of the protein was mainly found in the lining layer and was associated with vessels in the sublining layer. RT-PCR analysis showed *JAM-C *mRNA expression both in mouse synovial tissue samples and in cultured mouse synovial fibroblasts (Figure 2c).

### Antibodies against junctional adhesion molecule-C decrease the severity of murine antigen-induced arthritis

**Figure 3:**
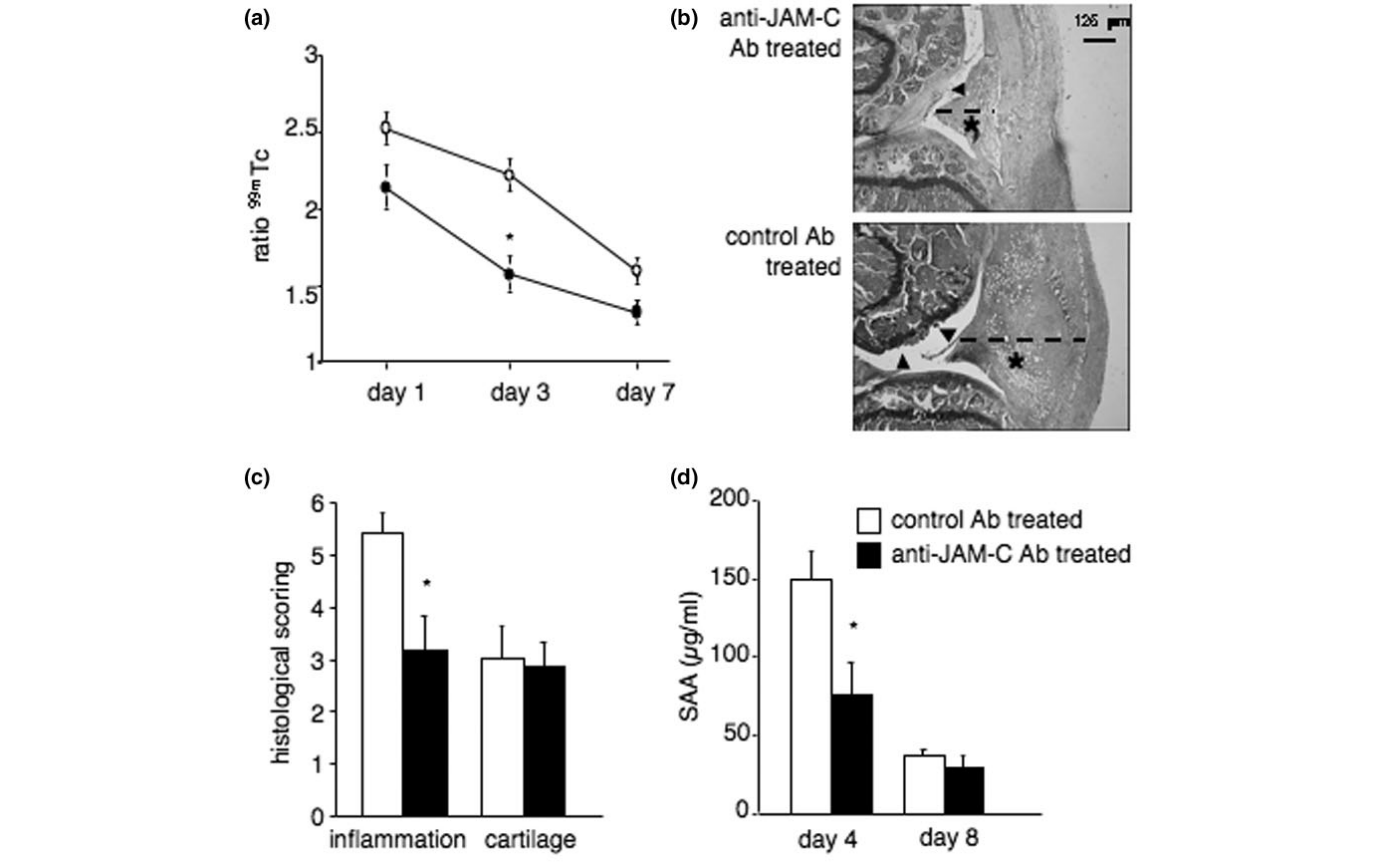
Treatment with anti-JAM-C antibody decreases the severity of antigen-induced arthritis. **(a) **Joint inflammation on days 1, 3, and 7 after intra-articular methylated bovine serum albumin (mBSA) injection. Results are expressed as the ratio of technetium-99m (^99m^Tc) uptake in the arthritic over the non-inflamed knee. The mean ± standard error of the mean (SEM) of the ratios is shown for anti-JAM-C antibody-treated (*n *= 10 for days 1 and 3, *n *= 6 for day 7; black symbols) and isotype-matched control antibody-treated (*n *= 10 for days 1 and 3, *n *= 5 for day 7; open symbols) mice. Joint inflammation was significantly reduced in anti-JAM-C antibody-treated mice on day 3. **(b) **Representative histological sections for anti-JAM-C antibody-treated (upper panel) and isotype-matched control antibody-treated (lower panel) mice 8 days after intra-articular mBSA injection (original magnification × 40, scale bar = 125 μm). Arrowheads: cartilage erosions; asterisks: pannus; broken lines: synovial thickness. **(c) **Histological scores shown as the mean ± SEM for anti-JAM-C antibody-treated (*n *= 6, black columns) and isotype-matched control antibody-treated (*n *= 5, open columns) mice 8 days after intra-articular mBSA injection. Synovial inflammation was significantly reduced in anti-JAM-C antibody-treated mice. **(d) **Circulating levels of serum amyloid A (SAA) on days 4 and 8 after intra-articular mBSA injection. Results shown represent the mean ± SEM for anti-JAM-C antibody-treated (*n *= 5 on day 4, *n *= 6 on day 8, black columns) and isotype-matched control antibody-treated (*n *= 5 at both time points, open columns) mice. Serum SAA levels were significantly decreased in anti-JAM-C antibody-treated mice on day 4. *p < 0.05 versus control mice, as assessed by analysis of variance. Ab, antibody; JAM-C, junctional adhesion molecule-C.

To investigate a potential role for JAM-C in arthritis, we tested the effect of the monoclonal anti-JAM-C antibody H36 [17] on the course of AIA. Injection of an isotype-matched control antibody did not affect the development of AIA as compared to treatment with PBS (data not shown). In contrast, treatment of mice with the anti-JAM-C antibody significantly decreased the severity of arthritis, as quantified by Tc uptake on day 3 (Figure 3a). Histology showed lower inflammation in knees of mice treated with the anti-JAM-C antibody as compared to isotype-matched control antibody-treated mice (Figure 3b,c) on day 8, whereas cartilage damage was similar in the two groups. The lack of effect of JAM-C antibody on cartilage might be accounted for, in part, by the lack of sensitivity of safranin O staining, which cannot detect subtle changes in cartilage degradation. Serum SAA levels, which reflect the systemic inflammatory response and are maximal on day 4, were significantly decreased in anti-JAM-C antibody-treated mice (Figure 3d).

### Antibodies against junctional adhesion molecule-C decrease the cellular immune response during antigen-induced arthritis

**Figure 4:**
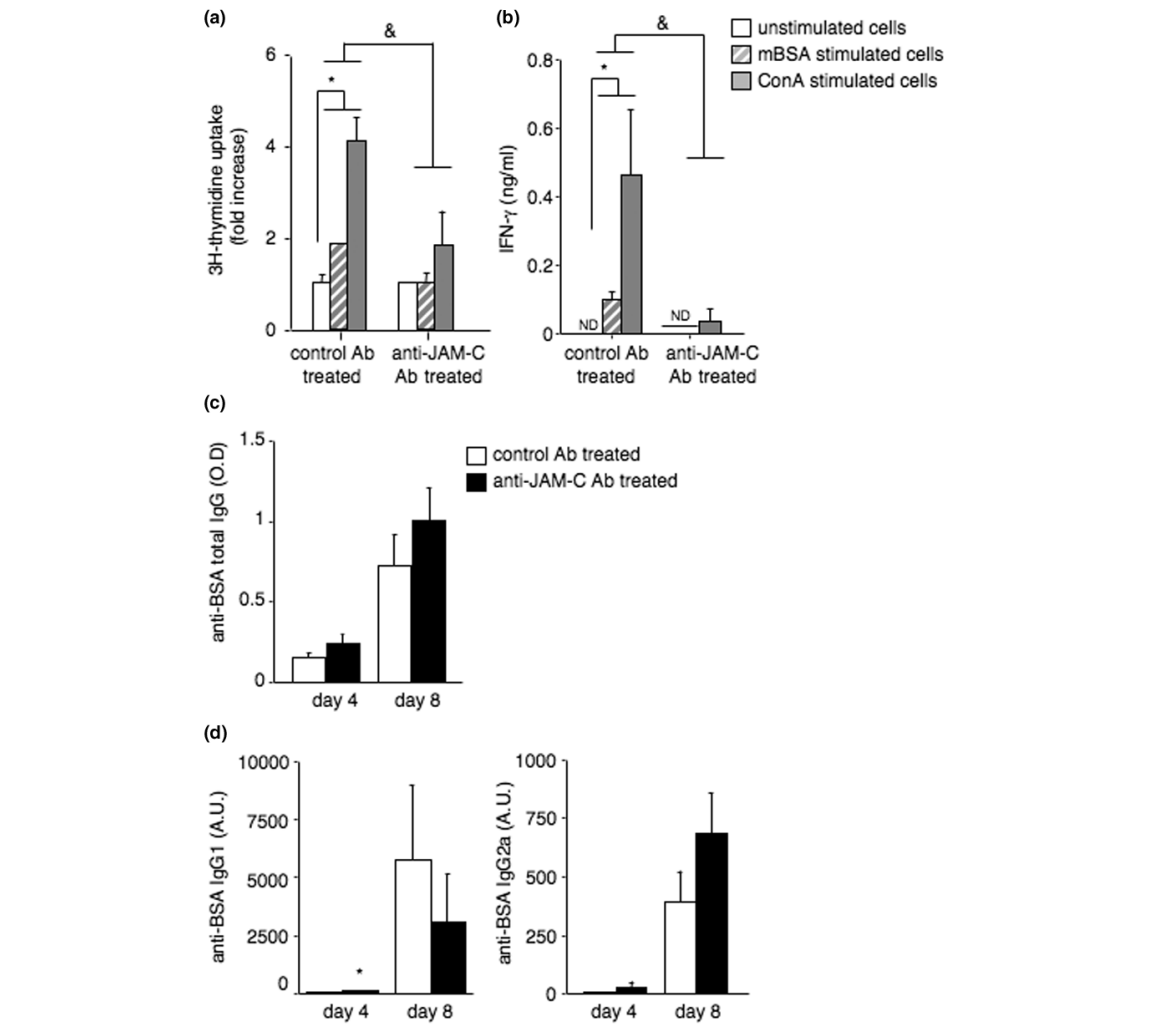
Treatment with anti-JAM-C antibody decreases the T-cell response. **(a) **Proliferation of spleen cells isolated from anti-JAM-C antibody-treated (*n *= 5) and isotype-matched control antibody-treated (*n *= 5) mice. Cells were restimulated *in vitro *with methylated bovine serum albumin (mBSA) (10 μg/ml, hatched columns) or concanavalin A (ConA) (5 μg/ml) or were left unstimulated (white columns). Results, expressed as fold increase in proliferation of stimulated over unstimulated cultures, represent the mean ± standard error of the mean (SEM) for each group of mice. Proliferation was significantly increased by stimulation with mBSA or ConA in cells isolated from isotype-matched control antibody-treated mice; *p < 0.05 versus unstimulated cultures. This proliferative response to mBSA and ConA was significantly reduced in cells of anti-JAM-C antibody-treated mice as compared to cells isolated from isotype-matched control antibody-treated mice; ^&^*p *< 0.05 versus cells isolated from isotype-matched control antibody-treated mice with similar stimulation, as assessed by analysis of variance (ANOVA). **(b) **Interferon-gamma (IFN-γ) production by spleen cells isolated from anti-JAM-C antibody-treated (*n *= 5) and isotype-matched control antibody-treated (*n *= 5) mice. Cells were restimulated *in vitro *with mBSA (10 μg/ml, hatched columns) or ConA (5 μg/ml) or were left unstimulated (white columns). Results shown represent the mean ± SEM for each group of mice. IFN-γ production was significantly increased by stimulation with mBSA or ConA in cells isolated from isotype-matched control antibody-treated mice; *p < 0.05 versus unstimulated cultures. This IFN-γ response to mBSA and ConA was markedly reduced in cells of anti-JAM-C antibody-treated mice as compared to cells isolated from isotype-matched control antibody-treated mice; ^&^*p *< 0.05 versus cells isolated from isotype-matched control antibody-treated mice with similar stimulation, as assessed by ANOVA. Serum levels of anti-mBSA total IgG **(c)**, IgG1 (**[d]**, left panel), and IgG2a (**[d]**, right panel) on days 4 and 8 after intra-articular mBSA injection. Results shown represent the mean ± SEM for anti-JAM-C antibody-treated (*n *= 6, black columns) and isotype-matched control antibody-treated (*n *= 5, open columns) mice. *p < 0.05 versus control mice, as assessed by ANOVA. Ab, antibody; A.U., arbitrary units; Ig = immunoglobulin; JAM-C, junctional adhesion molecule-C; O.D., optical density.

Proliferation of spleen cells isolated from mice treated with the isotype-matched control antibody was significantly increased upon restimulation with mBSA or ConA *in vitro *(Figure 4a). This proliferative response was significantly reduced in mice treated with the anti-JAM-C antibody (Figure 4a). Similarly, IFN-γ production was significantly increased by stimulation with mBSA or ConA in cells isolated from isotype-matched control antibody-treated mice, and this IFN-γ response was also markedly reduced in cells of anti-JAM-C antibody-treated mice (Figure 4b). IL-10 production was not detectable in spleen cell supernatants. A similar, though less marked, diminution of cell proliferation and IFN-γ production was also observed in draining lymph node cell cultures obtained from anti-JAM-C antibody-treated mice (data not shown). Lymph node cells obtained from isotype-matched control antibody-treated mice produced detectable levels of IL-10 upon stimulation with mBSA, and this IL-10 production was also reduced in cells isolated from anti-JAM-C antibody-treated mice. Total anti-BSA IgG and anti-BSA IgG2a levels were comparable in anti-JAM-C antibody-treated and isotype-matched control antibody-treated mice on days 4 and 8 (Figure 4c,d). Anti-BSA IgG1 levels were transiently elevated at day 4, but not at day 8, in the anti JAM-C antibody-treated mice.

**Figure 5:**
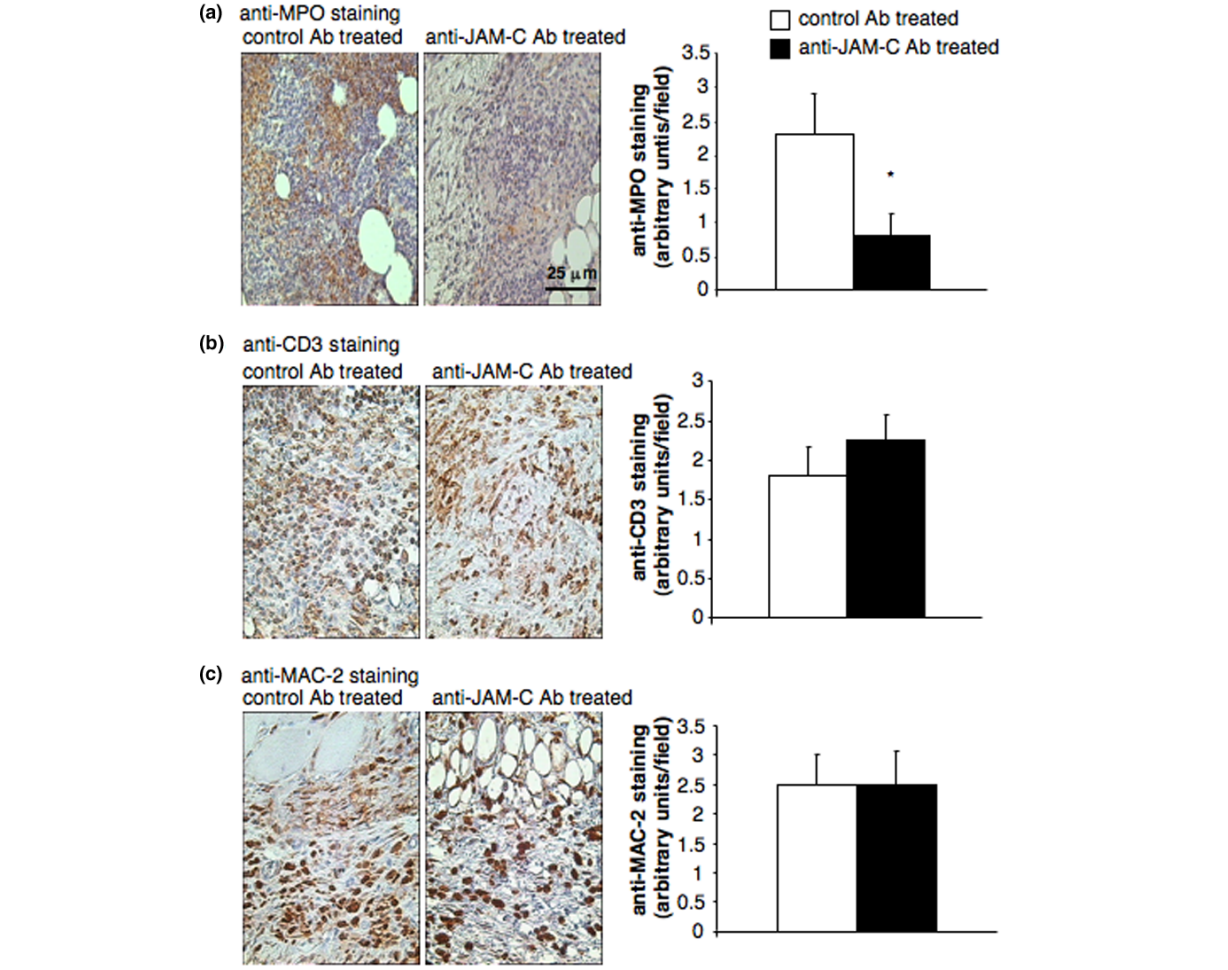
Treatment with anti-JAM-C antibody decreases neutrophil infiltration into the joints during antigen-induced arthritis. Infiltration of neutrophils **(a)**, lymphocytes **(b)**, and macrophages **(c) **into the synovium was detected by immunohistochemistry using anti-MPO, anti-CD3, and anti-MAC-2 antibodies, respectively. The left panels show representative knee joint sections of control and anti-JAM-C antibody-treated mice. Original magnification × 400 (scale bar = 25 μm). In the right panels, leukocyte infiltration per field was evaluated by semi-quantitative scoring for anti-JAM-C antibody-treated (*n *= 6, black columns) and isotype-matched control antibody-treated (*n *= 5, open columns) mice. There was a significant decrease in synovial neutrophil infiltration in anti-JAM-C antibody-treated mice as compared to isotype-matched antibody-treated controls. *p < 0.05 versus control mice, as assessed by analysis of variance. Ab, antibody; JAM-C, junctional adhesion molecule-C.

Neutrophil infiltration in the inflamed synovium was selectively reduced in anti-JAM-C antibody-treated mice, whereas lymphocyte and macrophage infiltrations per field were similar in anti-JAM-C antibody and isotype-matched control antibody-treated mice (Figure 5).

### Treatment with anti-junctional adhesion molecule-C antibody delays the onset of K/BxN serum transfer-induced arthritis

**Figure 6:**
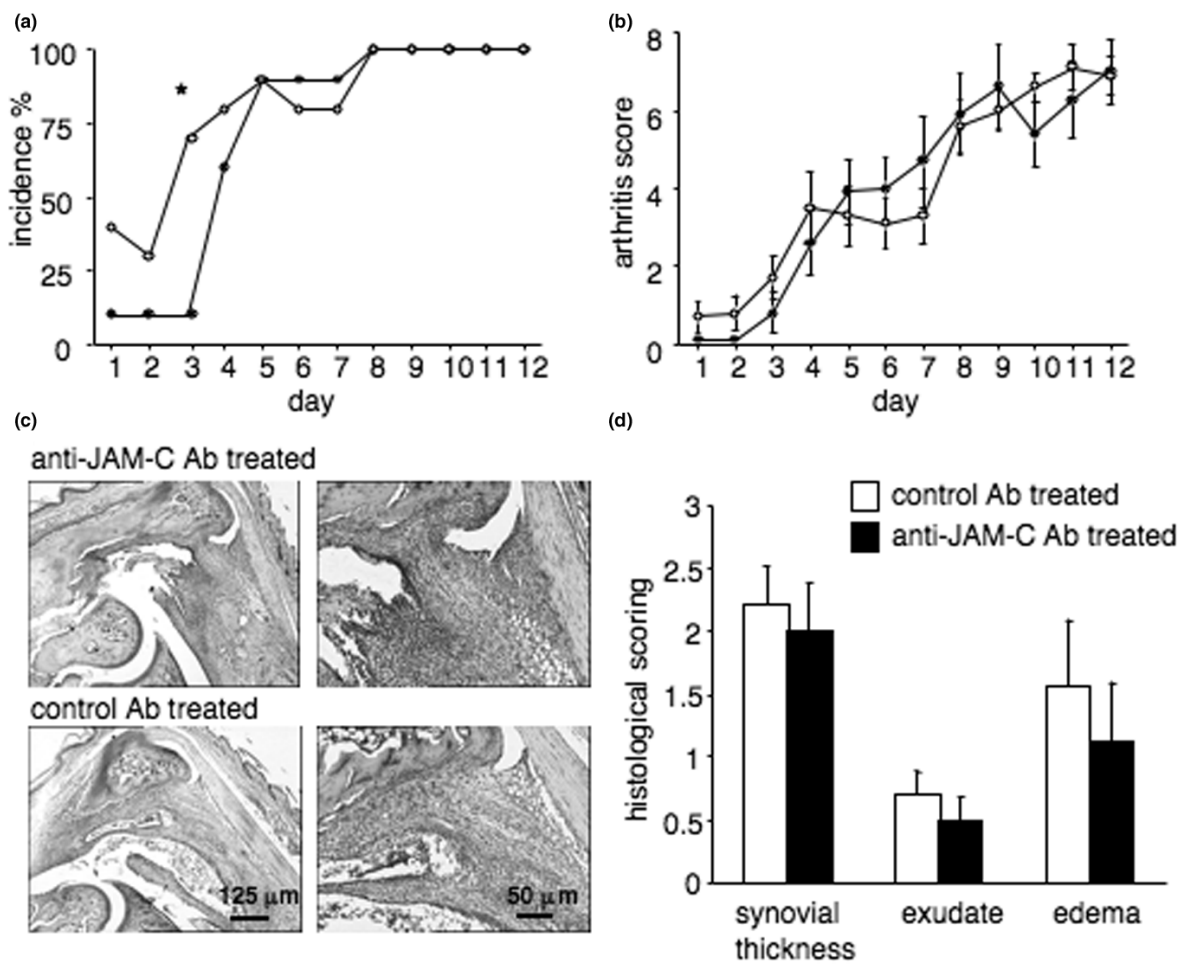
Treatment with anti-JAM-C antibody delays onset of K/BxN serum transfer-induced arthritis. **(a) **Incidence of K/BxN serum transfer-induced arthritis is shown for anti-JAM-C antibody-treated (*n *= 10, black symbols) and isotype-matched control antibody-treated (*n *= 10, open symbols) mice. Results are expressed as the percentage of arthritic mice per group. Incidence of arthritis was significantly lower in anti-JAM-C antibody-treated mice as compared to isotype-matched antibody-treated controls on day 3. *p < 0.05 versus control mice, as assessed by chi-square test. **(b) **Severity of K/BxN serum transfer-induced arthritis. Arthritis was evaluated by clinical assessment of arthritis severity scores for anti-JAM-C antibody-treated (*n *= 10, black symbols) and isotype-matched control antibody-treated (*n *= 10, open symbols) mice. Results shown represent the mean ± standard error of the mean (SEM) for each group of mice. There were no significant differences between the groups. **(c) **Histological assessment of arthritis. Representative sections are shown for anti-JAM-C antibody-treated (upper panels) and isotype-matched control antibody-treated (lower panels) mice 13 days after serum transfer. Original magnifications × 40 (left panel) (scale bar = 125 μm) and × 100 (right panel) (scale bar = 50 μm). **(d) **Histological scores for synovial thickness, exudates, and edema 13 days after serum transfer. Results shown represent the mean ± SEM for anti-JAM-C antibody-treated (*n *= 10, black columns) and isotype-matched control antibody-treated (*n *= 10, open columns) mice. No significant differences were observed between the groups. Ab, antibody; JAM-C, junctional adhesion molecule-C.

To specifically investigate a potential role of JAM-C in the effector phase of arthritis, we tested the impact of anti-JAM-C antibody treatment in a model of K/BxN serum transfer-induced arthritis. Treatment with the anti-JAM-C antibody delayed the onset of K/BxN serum transfer-induced arthritis, as indicated by a significantly lower incidence of arthritis in anti-JAM-C antibody-treated mice as compared to isotype-matched control antibody-treated mice on day 3 (Figure 6a). In contrast, the severity of arthritis was not affected by the treatment in this model (Figure 6b). We also verified that injection of the isotype-matched control antibody 9B5 *per se *had no effect on the development of serum transfer-induced arthritis (data not shown).

Histological analysis of the paws showed no difference between anti-JAM-C antibody-treated and control mice at day 13 (Figure 6c), although we cannot exclude the possibility that histological differences, and in particular differences in neutrophil infiltration, could have been detectable at an earlier time point. The serum SAA levels, which are not significantly increased in this model, were not affected by treatment with the anti-JAM-C antibody. In contrast, ConA-induced spleen cell proliferation *in vitro *was decreased in anti-JAM-C antibody-treated arthritic mice as compared to isotype-matched antibody-treated controls (fold increase in proliferation over unstimulated control cultures: 11.3 ± 0.67 for anti-JAM-C antibody-treated mice, *n *= 10; 37.1 ± 4.49 for control mice, *n *= 10; *p *< 0.05).

## Discussion

The protein JAM-C is an adhesion molecule involved in junctional interaction between adjoining endothelial, epithelial, or fibroblastic cells [7]. It has also been reported to regulate monocyte and neutrophil adhesion to and transmigration through various types of cells, including fibroblast, epithelial, and endothelial cells [6,11,22,23]. Although these results suggest that JAM-C is involved in leukocyte recruitment during inflammation, the role of JAM-C in human inflammatory diseases has not been well studied. Here, we found that JAM-C is present on vessels and synovial fibroblasts of human and mouse arthritic lesions. An antibody against JAM-C inhibits the recruitment of neutrophils to the joints during AIA and delays the onset of serum transfer-induced arthritis. Finally, treatment with anti-JAM-C antibody decreases the T-cell response in AIA.

Recruitment of inflammatory cells, maintenance of the inflammatory state, and the sustained retention of leukocytes within arthritic lesions are mandatory for the development of arthritis. The invasion of leukocytes into the synovial tissue is controlled at the molecular level by interactions between adhesion molecules expressed on endothelial cells and leukocytes [24]. The adhesion molecule JAM-C expressed by endothelial cells has been shown to bind the leukocyte integrin Mac-1 expressed by neutrophils and monocytes [6,8]. Although Mac-1 is not the only leukocyte adhesion molecule involved in leukocyte migration to arthritic lesions, this integrin, together with lymphocyte function-associated antigen-1 (LFA-1) and α4β1 integrins, has been shown to contribute to the development of the disease [25-27]. We know from previous studies that our anti-JAM-C monoclonal antibody does not prevent direct interaction between Mac-1 and JAM-C [15]. Nevertheless, it still blocks neutrophil recruitment to inflamed tissues [13]. One possibility is that treatment with the anti-JAM-C antibody modulates the contribution of Mac-1 or α4β1 integrins to leukocyte trafficking indirectly by interfering with the function of JAM-C [6,28]. Alternatively, the anti-JAM-C antibody may act directly on resident synovial fibroblasts to disrupt retention of granulocytes within lesions. Synovial fibroblasts are indeed potent producers of cytokines, adhesion molecules, and chemokines that attract and retain large numbers of leukocytes in the inflamed synovium, thus sustaining chronic inflammation and preventing the resumption of normal tissue homeostasis [29].

The mouse AIA model can be divided into two phases. Intra-articular antigen injection first results in an acute inflammatory reaction, characterized by joint swelling and leukocyte infiltration, which later proceeds to a chronic destructive arthritis with synovial hyperplasia, and cartilage and bone erosion. In addition to an inhibitory effect of anti-JAM-C antibody treatment on the acute inflammatory response, which is in line with previous findings [11-13], we observed an anti-inflammatory effect in the chronic phase of the disease, as shown by reduced inflammation in the knees of mice treated with anti-JAM-C antibody as compared to isotype-matched control antibody-treated mice on day 8 (Figure 3b,c).

The systemic suppressive effect of anti-JAM-C on T-cell response is an unexpected result. JAM-C is not expressed on mouse T cells and *in vitro *addition of anti-JAM-C antibodies onto spleen cell cultures did not alter cell proliferation (data not shown), suggesting that the immunomodulatory effect of the anti-JAM-C antibody may be mediated by stromal cells *in vivo*. Consistent with this observation, other investigators found no difference regarding the number of circulating lymphocytes between anti-JAM-C-treated and isotype control-treated groups in a model of allergic contact dermatitis responsive to anti-JAM-C administration [12]. We also observed that circulating levels of lymphocytes were not significantly decreased in naive and arthritic mice (K/BxN experiments) following the administration of the anti-JAM-C or the isotype-matched control antibody. The absolute lymphocyte counts (mean ± standard error of the mean) were 4.5 ± 0.57 g/L (naive anti-JAM-C antibody-treated), 5.5 ± 0.41 g/L (naive isotype-matched control antibody-treated), 6.89 ± 0.55 g/L (arthritic anti-JAM-C antibody-treated), and 5.5 ± 0.57 g/L (arthritic isotype-matched control antibody-treated). In addition, as in AIA, the inhibitory effect of anti-JAM-C antibodies occurred when mice were treated after sensitization and before challenge, thus indicating that JAM-C interferes with the effector phase and not the initial antigen presentation [12].

JAM-C is expressed on synovial and other types of fibroblasts, fibroblastic reticular cells of lymph nodes, and smooth muscle cells [10,23]. All these cells have in common an immunoregulatory function, and the role of fibroblastic cells in the establishment and maintenance of micro-environmental 'niches' contributing to inflammatory diseases, including RA, has been extensively discussed in recent reports [29-31]. Though speculative, one could imagine that anti-JAM-C treatment delivers signals to mesenchymal cells, which in turn control the outcome of the immune response by modulating expression of adhesion molecules, chemokines, and cytokines [32]. Such a hypothesis is in agreement with recent findings showing that anti-JAM-C treatment decreases inflammation in various mouse inflammatory models such as cerulein-induced pancreatitis, thioglycollate-induced peritonitis, or allergic contact dermatitis [11-13] and that the protein may be involved in other diseases such as obstructive nephropathy or atherosclerosis [10,33]. An alternative explanation for the systemic pro-inflammatory function of JAM-C relates to its active role in favoring diffusion of small molecules across the paravascular space. Indeed, we have previously shown that the enrichment of the protein at interendothelial cell-cell contacts correlates with increased paracellular permeability and is induced by factors inducing vascular leakage such as vascular endothelial growth factor (VEGF) [20,34]. These results have been confirmed in a recent study showing that antagonizing JAM-C function results in inhibition of increased vascular permeability induced by VEGF or histamine [35]. Thus, it is possible that treatment of arthritic mice with the anti-JAM-C antibody results in decreased diffusion of inflammatory mediators in the blood, inducing a systemic immunosuppressive effect. Although this attractive hypothesis requires more investigation, our results uncover a link between JAM-C expression in arthritic lesions and local and systemic anti-inflammatory effects of an antibody directed against this protein.

There is accumulating evidence that leukocyte trafficking to the inflamed synovium is adhesion molecule-dependent, and the blocking adhesion molecules that mediate the accumulation of leukocytes in inflammation can thus be expected to have therapeutic potential in human RA. However, the few clinical trials performed so far have not met expectations. For instance, efazulimab, a humanized monoclonal antibody against LFA-1, did not significantly reduce arthritis in a cohort of patients with psoriatic arthritis [36]. Anti-ICAM-1 (intracellular adhesion molecule-1) monoclonal antibodies have been evaluated in a phase I/II open-label study and had only limited effects in patients with RA [37]. Finally, a randomized, placebo-controlled trial of an antisense oligodeoxynucleotide ICAM-1 inhibitor could not demonstrate clinical efficacy beyond that of placebo in patients with active RA [38]. Among the possible explanations for this low efficacy are the redundancy and overlapping functions of molecules that are involved in leukocyte extravasation, due to which selective inhibition of single adhesion molecules might not be sufficient to efficiently prevent leukocyte recruitment. In the present study, we observed that treatment with an anti-JAM-C antibody induced anti-inflammatory effects, which seem to extend beyond local inhibition of leukocyte adhesion. This observation fits with the concept that the signaling activity of adhesion molecules, in addition to their adhesive properties, may be important for their function.

## Conclusion

JAM-C is highly expressed by synovial fibroblasts in RA. Treatment of mice with an anti-JAM-C antibody significantly reduced the severity of AIA and delayed the onset of serum transfer-induced arthritis, suggesting a role for JAM-C in the pathogenesis of arthritis.

## Abbreviations

AIA = antigen-induced arthritis; ConA = concanavalin A; DMEM = Dulbecco's modified Eagle's medium; ELISA = enzyme-linked immunosorbent assay; ICAM-1 = intracellular adhesion molecule-1; IFN-γ = interferon-gamma; Ig = immunoglobulin; IHC = immunohistochemistry; IL-10 = interleukin-10; i.p. = intraperitoneal; JAM = junctional adhesion molecule; LFA-1 = lymphocyte function-associated antigen-1; mBSA = methylated bovine serum albumin; OA = osteoarthritis; PBS = phosphate-buffered saline; PCR = polymerase chain reaction; PECAM-1 = platelet endothelial cell adhesion molecule-1; RA = rheumatoid arthritis; RT = reverse transcriptase; SAA = serum amyloid A; Tc = technetium; VEGF = vascular endothelial growth factor.

## Competing interests

The authors declare that they have no competing interests.

## Authors' contributions

GP, NB, and MA-L performed the experiments, designed the study, and drafted the manuscript. DT-A, VC-P, CZ, and PH performed the experiments. BAI and CG designed the study and drafted the manuscript. All authors read and approved the final manuscript. GP and NB contributed equally to this work.
